# Genetic diversity of porcine circoviruses 2 and 3 circulating among wild boars in the Moscow Region of Russia

**DOI:** 10.3389/fvets.2024.1372203

**Published:** 2024-06-26

**Authors:** Nikita Krasnikov, Valentina Rykova, Oksana Kucheruk, Alina Komina, Alexander Pchelnikov, Alexey Gulyukin, Anton Yuzhakov

**Affiliations:** Laboratory of Biochemistry and Molecular Biology, Federal State Budget Scientific Institution “Federal Scientific Center VIEV” (FSC VIEV), Moscow, Russia

**Keywords:** PCV2, PCV3, wild boar, complete genome, phylogenetics, wildlife, epitopes

## Abstract

Porcine circoviruses (PCVs) are widely distributed in swine herds. PCV2, the significant swine pathogen, causes infections characterized by growth and development disorders, skin lesions, and respiratory distress. PCV3 has been circulating worldwide and can be associated with various clinical signs and disease developments. Wild boars are the main reservoir of these pathogens in wildlife and can create an alarming threat to pig farming. In Russia, three PCV2 genotypes (PCV2a, PCV2b, and PCV2d) were identified in pig farms. Additionally, PCV3 was observed in pig herds during the monitoring studies in the country. However, data considering the circulation of PCVs in herds of wild boars in Russia is scant. For this purpose, we performed PCR assays of the samples from 30 wild boars hunted in the Moscow Region of Russia in 2021–2023. The ratios of wild boars positive for PCV2, PCV3, or coinfected were 50, 10, and 13.3%, respectively. Additionally, we sequenced 15 PCV2 and four PCV3 complete genomes and conducted phylogenetic analysis, which divided PCV2 isolates into two groups: PCV2d and PCV2b. The study showed a high infection rate of PCV2 among wild boars, with PCV2d dominance. Simultaneously, PCV3 also circulates among wild boars. The obtained results can provide a basis for the development of preventive measures to support infection transmission risks between farm and wild animals.

## Introduction

Porcine circovirus (PCV) was originally identified as a small icosahedral, non-enveloped virus 17 nm in diameter with a circular ssDNA genome size of 1.7 kb (1). Currently, porcine circoviruses are members of the family *Circoviridae*, genus *Circovirus*, which comprises four species (PCV1-4).

PCV1 was primarily detected in 1974 as a contaminant of the permanent PK-15 cell line (2) and is currently considered non-pathogenic (3). In contrast, PCV2 is an emerging swine pathogen. It was first isolated from pigs with post-weaning multisystemic wasting syndrome (PMWS) and actively studied in the 1990s and 2000s (4). The virus is linked to the porcine circovirus-associated disease (PCVAD) (5). PCVAD can manifest itself in various ways and includes PMWS (6), porcine dermatitis and nephropathy syndrome (PDNS) (7), pneumonia (8), myocarditis (9), reproductive failure and abortions (10), enteritis (11), hepatitis (12), and congenital tremors (13). PCV2 is a ubiquitous global pathogen. Retrospective studies showed that the virus has been detected in samples from pigs since the 1960s in different countries (14, 15).

Presently, among all known porcine circoviruses, the genome structure has been studied more extensively for PCV2. It is assumed that PCV2 genomic DNA contains 11 open reading frames (ORFs) and at least seven potential ORF-coding proteins. But the existence of only four ORFs encoding five proteins has been proven now: ORF1 coding Rep (replication-associated protein) and Rep′ (spliced frame-shifted version of Rep), ORF2 coding capsid protein (Cap), ORF3 coding apoptotic protein, and ORF4 coding unidentified anti-apoptotic protein, respectively (16). Due to its high genetic variability, ORF2 is usually used for phylogenetic analysis.

PCV3 was first detected in 2015 in the United States in sows with PDNS and in their aborting piglets (17). Since it was discovered, data on its prevalence around the world has appeared (18–20). PCV3 was found both in healthy and diseased pigs. Its pathogenicity is not obvious yet; however, the virus is often associated with reproductive failures (21), PDNS (17), multi-systemic disease, and cardiac pathology (22).

PCV4 was first registered in China in 2019 (23). Since then, this novel virus has been detected in domestic pigs in Thailand (24), South Korea (25), and more recently, in wild boars and domestic pigs in Europe (26). Simultaneously, its pathogenicity remains unclear.

Porcine circoviruses are an enormously genetically diverse group of viruses. Pathogenic PCV2 has eight distinct genotypes (a˗h) (27). Moreover, PCV3 and PCV4 can also presumably be divided into several genotype groups (24, 28).

In 1986, antibodies to PCV were first identified in wild boar’s samples (3). After that, wild boars have become considered a potential reservoir of PCVs and started to be studied widely worldwide. The following studies demonstrated PCV2 circulation among wild boars in North America (29), Brazil (30), Asian regions (31), and numerous European countries (32–34). Both PCV2 and PCV3 were found in wild boar populations in Japan (35), Italy (36), and China (37).

It should be assumed that the lack of information about PCV3 circulation in the wild boar population is associated solely with less interest in it than in PCV2, the pathogenicity and economic significance of which have been proven.

Both emerging porcine circoviruses, PCV2 and PCV3, have been detected in domestic pigs in Russia (20, 38). Meanwhile, there is no reliable data about the circulation of PCV2 and PCV3 in the wild boar population in the country. In connection with the above, we set out to conduct a study of the PCV2 and PCV3 presence and genetic diversity in the wild boar population in the Moscow Region of Russia.

## Materials and methods

### Sampling

From fall 2021 to winter 2023, specimens were sampled from 30 free-living wild boars (*Sus scrofa*). Sampling was performed during the autumn-winter hunting seasons. The animals originated from eight districts of the Moscow Region. The animals were hunted according to the special license in the regulated hunting area under the regulation of the special services. From each hunted animal, three different sample types were taken: bronchial lymph node, lung, and spleen. For all animals, the location and date of collection were recorded. Whenever possible, the age and gender were registered. The age was assessed by tooth eruption and replacement pattern. Information regarding clinical signs of infection and animal health status was not available. Collected samples were transported to the laboratory under freezing conditions.

### DNA extraction and PCR

From each organ sample, a piece of tissue was taken and homogenized in 50 mL Falcon tubes containing 5 mL of saline solution. Following that, the prepared samples were aliquoted in 1.5 mL Eppendorf tubes and stored at −70°C. The total DNA was extracted from the supernatant of organ suspension using the commercial kit “RIBO-prep” (FBIS Central Research Institute of Epidemiology of Rospotrebnadzor, Moscow, Russia) following the manufacturer’s instructions.

To detect PCV2 genome, real-time PCR (qPCR) was conducted using a commercial PCR test kit (Vetbiochem, Moscow, Russia) according to the manufacturer’s instructions. The samples with Ct ≤ 35 were considered positive. For PCV3 detection, PCR targeting the 330-bp internal cap gene was performed using primers developed by Palinski et al. (17). The PCR products were analyzed by electrophoresis in 1% agarose gel prepared in a Tris-acetate buffer solution (pH 8.0) with the addition of ethidium bromide (0.5 μg/mL) and the following gel examination under ultraviolet light.

### Complete genome sequencing and phylogenetic analysis

One sample with the best Ct-value in qPCR in the case of PCV2 or the best quality during electrophoresis in the case of PCV3 from each positive wild boar was subjected to complete genome sequencing and genotyping for further phylogenetic analysis.

To obtain complete genome sequences of PCV2 and PCV3, previously designed primers were applied (20, 38). In both cases, eight pairs of primers flanked four overlapping regions around 400–600 nt in length. The resulting amplified fragments covered the entire genome of the viruses. Further, PCR products were purified from gel using the Cleanup Standard Kit (Evrogen, Moscow, Russia) or the LumiPure Gel Extraction Kit (Lumiprobe, Russia). Purified samples were stored at −20°C until further analysis. The obtained fragments were sequenced in both directions using the BigDye Terminator v3.1 Cycle Sequencing kit on the ABI Prism 3,130 Genetic Analyzer (Thermo Fisher Scientific, Carlsbad, CA, United States). DNA sequence chromatograms were analyzed and assembled into final consensus using SeqMan Lasergene 11.1.0. (DNASTAR, Madison, WI, United States).

The phylogenetic analysis was performed using MEGA 7.0 software (39), with further visualization of Newick files using iTOL v6 (40). The obtained sequences were aligned by the MUSCLE algorithm. Phylogenetic dendrograms were plotted using the maximum likelihood (ML) method and the GTR (G + I) model. The topology evaluation was performed by 1,000 bootstrap replications. The pairwise genetic distances were calculated according to the Tamura 3-parameter model.

### Recombination analysis

Recombination events were evaluated using the Recombination Detection Program (RDP; v. 4.101) (41). For recombination between PCV2 from wild boars and PCV2 from domestic pigs, 15 complete genomes of isolates from this study (2 PCV2b, 13 PCV2d) and other 14 PCV2 isolates from Russia obtained earlier were applied (38). The aligned sequences were evaluated by the RDP, GeneConv, SiScan, MaxChi, BootScan, Chimera, and 3Seq algorithms.

## Results

### Epidemiology of PCVs in wild boars of the Moscow Region

Overall, 90 samples from 30 free-living wild boars were tested for the presence of PCV2 and PCV3 genomes. There were 10 males (33.3%), six females (20%), and 14 animals with unknown gender (46.7%). The samples originated from eight districts of Moscow Region in the following quantities: Solnechnogorsky district supplied the largest number (*n* = 9), followed by Taldomsky (*n* = 5), Yegoryevsky (*n* = 4), Orekhovo-Zuevsky (*n* = 4), Klinsky (*n* = 2), Dmitrovsky (*n* = 4), and one sample from Pavlovo-Posadsky and Ruzsky districts each.

**Figure 1:**
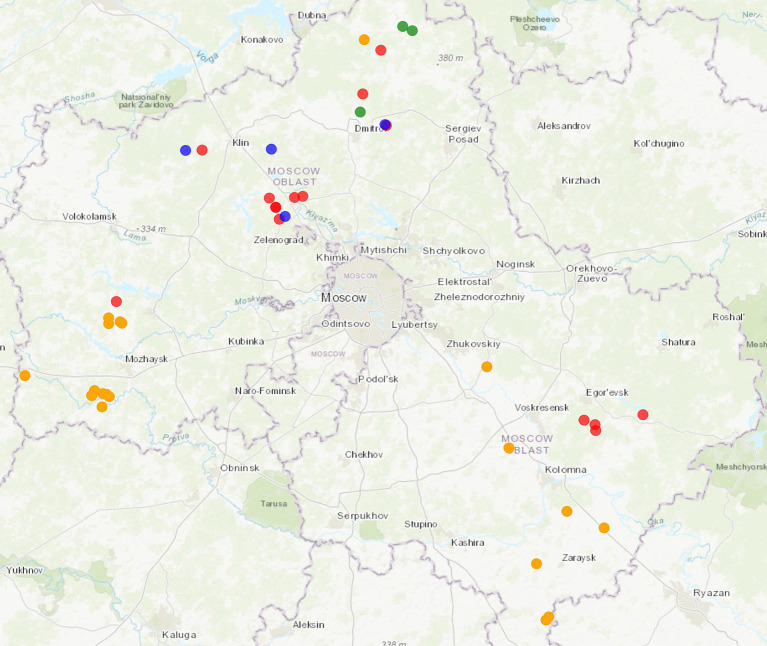
Map showing area in which wild boars with PCV2 and PCV3 were hunted in the Moscow Region. Colored dots are specified as follows: orange: intensive pig farming; red: PCV2-infected animals; green: PCV3-infected animals; blue: coinfected animals. The map was constructed using htmlwidgets, webshot, and leaflet R packages. The link to the detailed interactive web map: https://rpubs.com/krasnikovn/pcv_wildboars21-23.

According to the PCR test results, 15 animals were PCV2-positive, three were PCV3-positive, and four animals were coinfected. PCV-positive animals were predominantly identified in the north and south-east of the Moscow Region (Figure 1).

PCV2 genome was mainly detected in lymph node samples (38.3%), while PCV3 was prevalent in spleen samples (60%). The detailed information regarding the samples obtained in this study is available in the Supplementary Tables 1, 2.

### Genotyping and phylogenetic analysis of PCVs

Among PCV-positive wild boars, 15 complete PCV2 genomes and four complete PCV3 genomes were successfully sequenced for further genotyping. From one PCV2-positive wild boar (wild boar 10), only the ORF2 sequence was obtained. To our regret, there were some samples from which genomes were not sequenced because of the low virus load. The nucleotide sequences of the PCV isolates were deposited in the NCBI GenBank database under accession numbers OR960655-OR960674.

The genome sizes of 14 PCV2 and four PCV3 isolates identified in this study were 1767 and 2000 nt, like the majority of deposited PCV2 and PCV3, respectively. Noteworthy, one PCV2 sequence length (PCV2/RUS/2023/wild_boar_28) was 1766 nt, with a deletion in the 1744th position.

**Figure 2:**
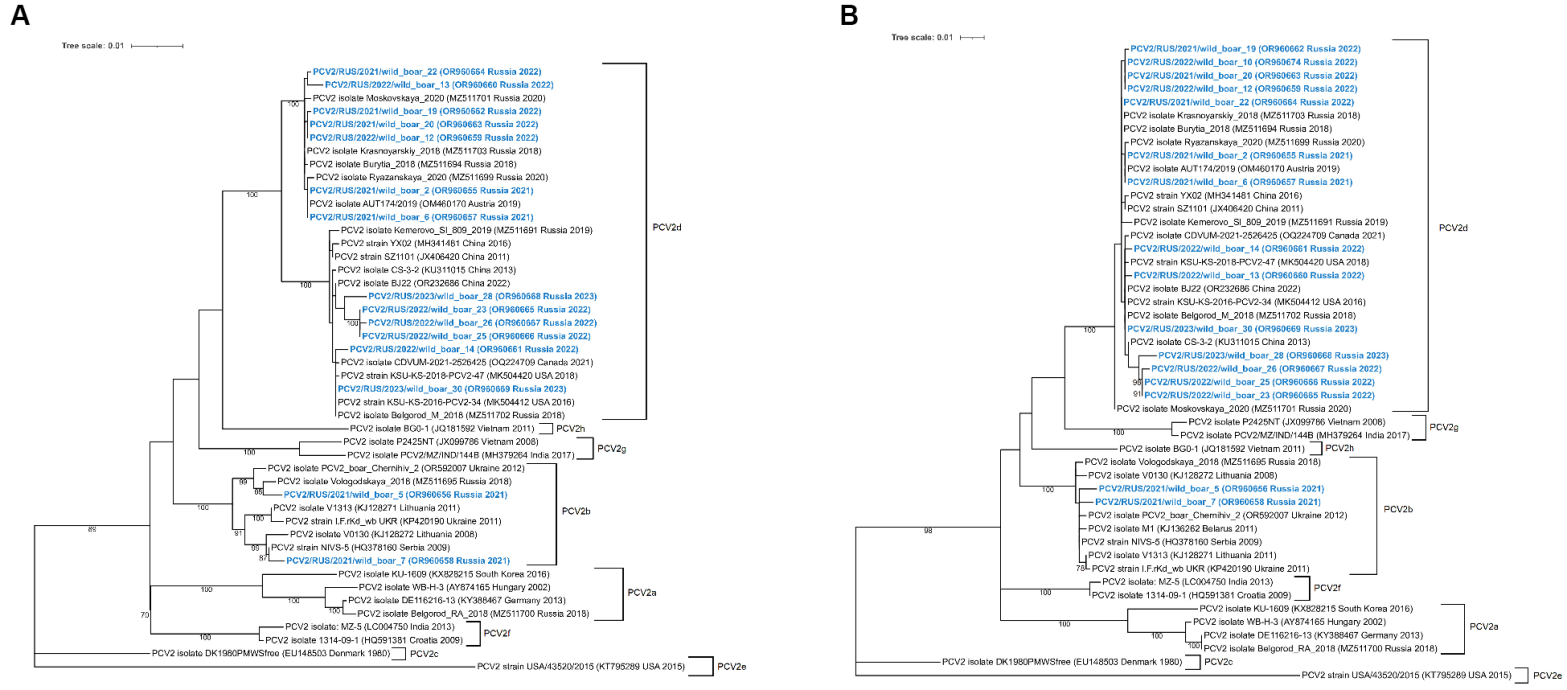
Complete-genome **(A)** and ORF2-based **(B)** phylogenetic dendrograms of PCV2 sequences constructed using the maximum likelihood method. A total of 1,000 bootstrap replications were used to estimate the robustness of the individual nodes on the phylogenetic dendrograms. Bootstrap values greater than 70% are specified. PCV2 isolates obtained in this study are colored in bold blue.

The constructed ML phylogenetic dendrograms based on the complete genome and ORF2 sequences revealed that PCV2 isolates were eventually divided into two genogroups: PCV2b (2/16) and PCV2d (14/16; Figure 2). The nucleotide pairwise identity between the PCV2 complete genome sequences varied between 95.4–100%. Both PCV2b isolates were identified in 2021 in one district of the Moscow Region. According to the complete genome analysis, the isolate PCV2/RUS/2021/wild_boar_5 was close to the isolate Vologodskaya_2018 (MZ511695), which was detected earlier on a pig farm in the Northwest Russia. Another PCV2b isolate (PCV2/RUS/2021/wild_boar_7) was similar (99.72%) to the strain NIVS-5 (HQ378160) from Serbia.

All of the obtained PCV2d isolates were divided into three separate clades. The first clade was quite close to other Russian isolates from domestic pigs, with a nucleotide identity in the range of 99.5–99.9%. It is remarkable that the two isolates from the first clade (PCV2/RUS/2021/wild_boar_2 and PCV2/RUS/2021/wild_boar_6) were identical to each other and the Austrian isolate AUT174/2019 (OM460170). The second clade included sequences that had a high degree of resemblance to strains from North America, and the third was formed with sequences close to the Chinese isolates.

**Figure 3:**
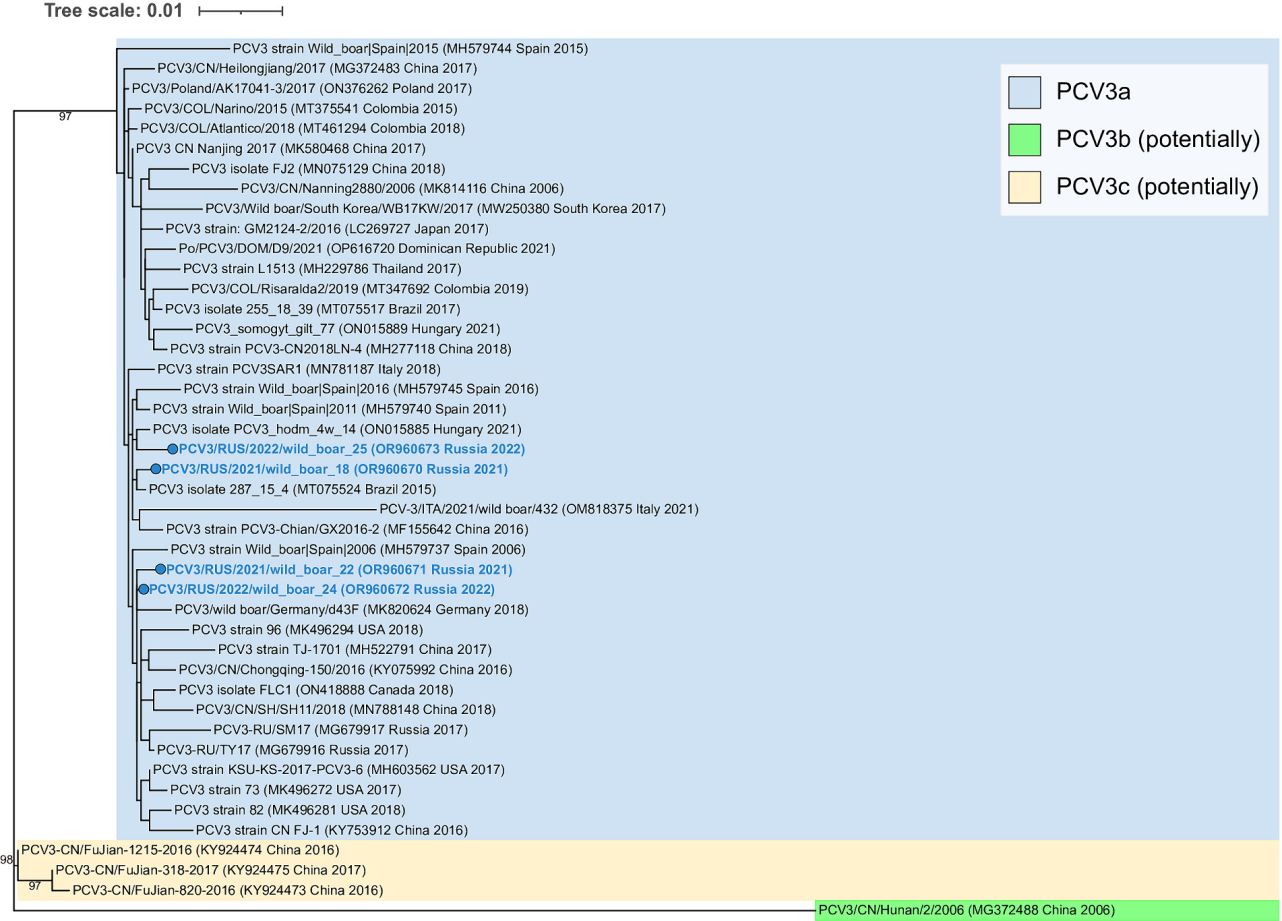
Complete-genome phylogenetic dendrogram of PCV3 sequences constructed using the maximum likelihood method. A total of 1,000 bootstrap replications were used to estimate the robustness of the individual nodes on the phylogenetic dendrogram. Bootstrap values greater than 90% are specified. PCV3 isolates obtained in this study are colored in bold blue.

All obtained PCV3 isolates were related to PCV3a genotype group (Figure 3). The sequences were similar to each other, and pairwise identity varied between 99.4–99.6%.

### Recombination analysis of the PCV2 isolates

According to all applied parametric tests, recombination analysis revealed no inter-or intragenotype recombination events between the PCV2 isolates from wild boars and previous isolates obtained from domestic pigs in Russia.

### Analysis of the main immunodominant epitopes on the PCV2 capsid protein

A comparison analysis of the main immunodominant epitopes on the PCV2 capsid protein between the PCV2a vaccine strain (AF264042) and the isolates obtained in this study was performed.

**Figure 4:**
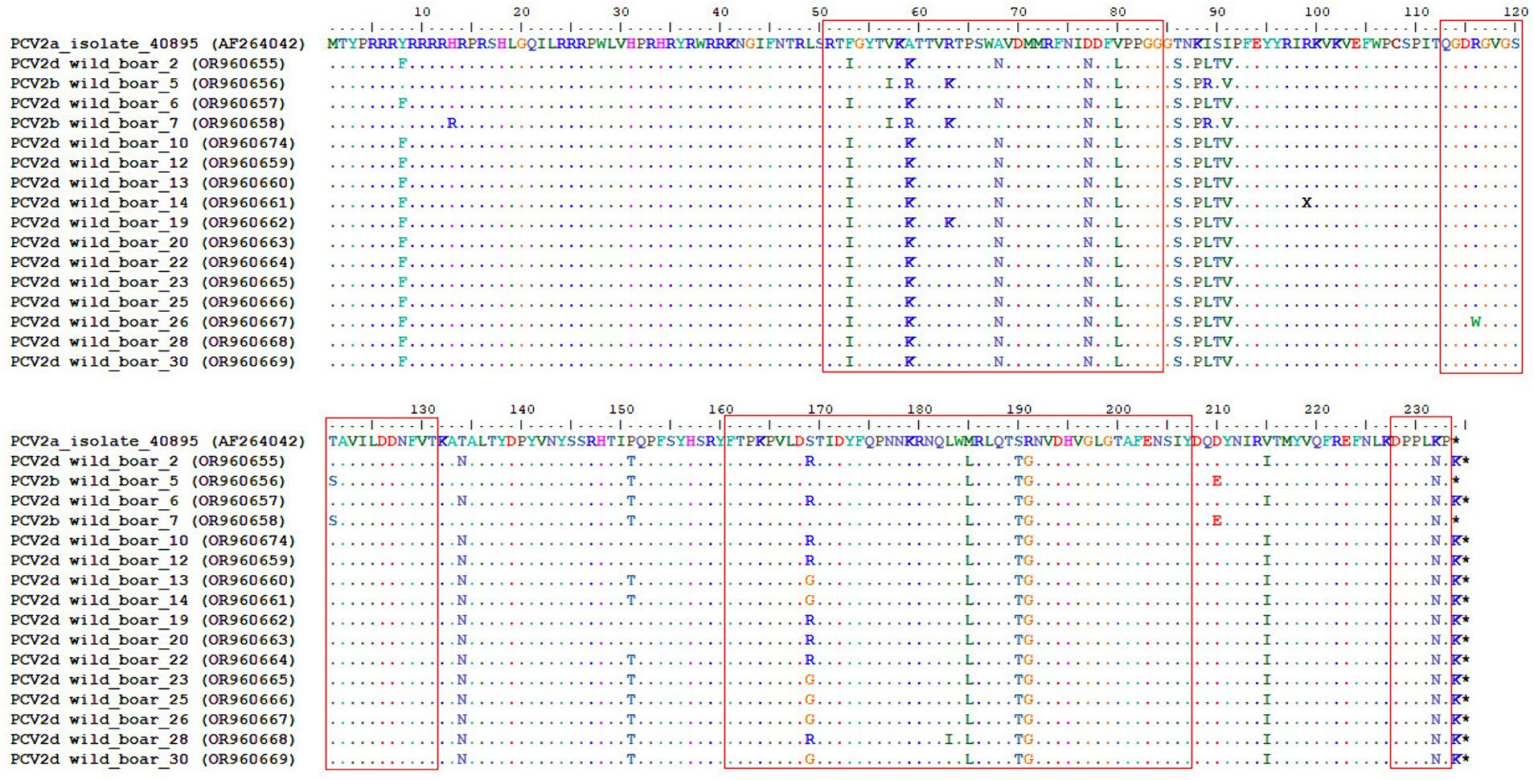
Complete alignment of the CAP protein amino acid sequences of the obtained PCV2 isolates. The top line corresponds to the PCV2a strain present in a commercial vaccine. Dots and hyphens represent identical amino acid positions and gapped positions, respectively. Asterisks represent stop codons. Antibody recognition domains [A (51–84), B (113–131), C (161–207), and D (228–233)] are highlighted with red boxes.

The nucleotide sequences of the ORF2 were aligned using the ClustalW method in the BioEdit software. A comparison of the capsid protein sequences between the vaccine strain revealed amino acid substitutions in all four known antibody recognition domains. The largest number of them was noted in the A (51–84) and C (161–207) antibody recognition domains (Figure 4).

## Discussion

Porcine circoviruses are abundant in pigs of all ages, resulting in economic losses to the pork industry worldwide. The wild boar is a principal potential reservoir of these viruses in wildlife. Previous seroprevalence and genetic characterization studies of PCV2 and PCV3 in wild boars have been reported in Europe, North and South America (29, 30, 32, 33). Recent research in Southern Italy showed widespread circulation of PCVs in the wild boar population, where the PCV2/PCV3 genome was detected in roughly 74% of analyzed samples both in the Campania and the Basilicata Regions (36, 42). A large-scale monitoring study among wild boars in China revealed the positive rates of PCV2 and PCV3 in 57.07% (113/198) and in 36.36% (72/198) of the cases, respectively, and the PCV2 and PCV3 co-infection rate was 19.7% (39/198) (37). In Asian countries, the circulation of PCV2 and PCV3 in wild boars was also observed in South Korea and Japan (31, 35). In our consideration, the positive rates of PCV2 and PCV3 were 50% (15/30) and 10% (3/30), and the PCV2 and PCV3 co-infection rate was 13.3% (4/30). The total positivity rate of PCV2/PCV3 cases equaled 73.3% (22/30).

It is also important to mention that the choice of sample type influences the final prevalence of PCVs. Certain studies showed that the detection of the PCV genome is more effective in tissue samples than in serum (43). The choice of the tissue sample also plays a key role in the virus detection. The study, performed in the Campania Region of Italy, showed that the frequencies of detection of PCV2 and PCV3 were found to be comparable in the brain and heart, whereas in the liver and spleen, PCV2/PCV3 frequencies were higher (liver 65.4%; spleen 72.2%) (42). Our results generally display a comparable picture of PCV circulation among wild boars and correspond to global prevalence rates.

The wild boar population in the Moscow Region of Russia is not indeed large but stable, with estimates of around 550 animals (44). During the analysis, PCV-positive animals were partially located nearby the intensive pig farming areas in the west and north of the studied region. In the Moscow Region, the majority of pig farms are large-scale with high biosecurity level, but there are still many holdings with obsolete protection and control measures. The wild boars visiting areas bordering pig farms with low biodefence systems increase the risks of pathogen introduction to domestic animals.

Of successfully sequenced isolates, PCV2b was verified in two of the instances, while PCV2d was linked to the remaining cases. It is noteworthy that there was no PCV2a among the genotyped isolates. PCV3 was noted in seven cases, including four in co-infections with PCV2. All currently known PCV3 strains are related to the PCV3a genotype. Recently, Franzo et al. have proposed to distinguish two additional groups: PCV3b and c, but there is still not enough sequencing data to be confident in this taxonomic separation (28). According to the constructed phylogenetic dendrogram, PCV3 isolates described in this study were strongly placed in the core of the PCV3a genogroup, and any connection between genetic diversity and geographical distribution was not observed.

Despite its modest genome size, PCV2 has the highest evolution rate among DNA viruses (45). PCV2a, the first detected genotype, was the prepotent from the late 1990s to 2000 and was then superseded by PCV2b. During the last decade, the second genomic shift from PCV2b to PCV2d has occurred worldwide, and now PCV2d has become the prevalent one (46). Actual data regarding PCV2 circulation in Russia is scarce. According to one study, PCV2 was already circulating in the wild boar population in 2002–2005 across several regions of Russia, including the Moscow Region (47). The whole genome study of the isolates obtained from 13 pig farms in Russia during 2018–2020 indicated the PCV2d prevalence in the country regions (38). Among countries bordering Russia, the monitoring study among wild boars in Ukraine in 2012 revealed a positive rate of PCV2 in 31.8% (34/107) of the samples with a PCV2b prevalence (9/10) (48). This finding can be explained by the fact that the samples were collected more than 10 years ago, when the PCV2b genotype ‘gained the upper hand.’ In this study, the significant dominance of the PCV2d genotype among the obtained isolates was confirmed in wildlife in the Moscow Region. Such global genetic change leads to the appearance of a burning question concerning the reliability of the currently available commercial vaccines.

The vast majority of the preventive vaccines against PCV2 are inactivated and based on the PCV2a genotype or its capsid protein. There is no unanimous opinion on the issue of the vaccine’s versatility against different PCV2 genotypes. The vaccines are effective in reducing clinical signs and production losses; however, their efficacy against the mixed infection of PCV2a and PCV2b and the currently prevalent PCV2d is controversial. According to several studies, PCV2a-based vaccines can be effective against the PCV2b and PCV2d challenges, reducing viremia, tissue loads, shedding, and transmission between the animals (49, 50). However, in the experimental work by Opriessnig et al., one out of nine vaccinated and experimentally PCV2-infected pigs developed clinical signs of PDNS (51). Nevertheless, the authors suggested that excessive PCV2 antibody titers may trigger the development of PDNS. Park et al. confirmed the presence of antibody cross-protection and reduced viremia to genotypes b and d when vaccinated with a PCV2a vaccine, but the titers of neutralizing antibodies were lower for PCV2b and PCV2d than for PCV2a (52). Such neutralizing antibody responses may originate due to amino acid differences in the neutralizing epitopes of the capsid protein among distinct PCV2 genotypes.

The capsid protein, coded for by ORF2, is the main target of antibody recognition. Some authors identified different numbers and positions of epitopes. Trible et al. determined four antibody recognition domains labeled A (51 to 84), B (113 to 131), C (161 to 207), and D (228 to 233) (53). The most essential positions for antibody recognition are Y-173, F-174, Q-175, and K-179. In our study, all of these critical amino acids were conservative and homogeneous. The most diverse was the A domain (51 to 84), which had the largest number of amino acid substitutions in the compared sequences with the vaccine strain.

Even with the mentioned vaccine benefits and its common usage, PCV2 infection is still widespread among vaccinated domestic pigs in different countries, including Russia (38). Most often, PCVAD outbreaks are associated with an incorrect vaccination plan when breaches in immune defense occur (54). However, PCV2 has also been isolated from clinically healthy vaccinated pigs, indicating that the virus proceeds to circulate in the herd despite the vaccination.

This actual study reveals the presence of PCV2 and PCV3 in wild boar herds in Russia. Our study demonstrates that PCV2d is the predominant genotype and PCV3 is circulating in wildlife in the Moscow Region. As Russia is located in both Europe and Asia and borders with plenty of other countries, including China, where the high genetic variability of PCV species was confirmed (55), it is necessary to regularly conduct such studies and obtain more genetic data regarding PCV diversity.

## Data availability statement

The data presented in the study are deposited in the NCBI GenBank repository, accession numbers OR960655-OR960674.

## Ethics statement

The animal study was approved by Ethical and Animal Welfare Committee of the Federal State Budget Scientific Institution “Federal Scientific Center VIEV,” (Moscow, Russia). Approval number 662/22 from 17 June 2021. The study was conducted in accordance with the local legislation and institutional requirements.

## Author contributions

NK: Conceptualization, Data curation, Formal analysis, Software, Validation, Visualization, Writing – original draft, Writing – review & editing. VR: Formal analysis, Investigation, Methodology, Writing – original draft. OK: Data curation, Investigation, Methodology, Writing – review & editing. AK: Investigation, Methodology, Writing – review & editing. AP: Resources, Writing – review & editing. AG: Funding acquisition, Project administration, Writing – review & editing. AY: Conceptualization, Data curation, Formal analysis, Funding acquisition, Supervision, Validation, Writing – review & editing.
