# Supplementary material for: Genetic diversity of porcine circoviruses 2 and 3 circulating among wild boars in the Moscow Region of Russia
Source: Front Vet Sci. 2024 Jun 26;11:1372203. doi: 10.3389/fvets.2024.1372203 (PMC11233533; doi:10.3389/fvets.2024.1372203)
Supplement: Supplementary file 2 [file Table_2.DOCX]

**Supplementary Table 2.** Numbers and their percentages of PCV-positive samples.

| Sample | PCV2,  N. infected | PCV2,  % infected | PCV3,  N. infected | PCV3,  % infected |
| --- | --- | --- | --- | --- |
| Lymph node | 18/30 | 38.3 | 2/30 | 20 |
| Spleen | 13/30 | 27.7 | 6/30 | 60 |
| Lung | 16/30 | 34.0 | 2/30 | 20 |
